# Psychological factors affecting COVID-19 vaccine acceptance in Indonesia

**DOI:** 10.1186/s41983-021-00436-8

**Published:** 2021-12-20

**Authors:** Theo Audi Yanto, Gilbert Sterling Octavius, Rivaldo Steven Heriyanto, Catherine Ienawi, Haviza Nisa, H. Emildan Pasai

**Affiliations:** 1 Department of Internal Medicine, Faculty of Medicine, Universitas Pelita Harapan, Karawaci, Tangerang, Banten Indonesia; 2 Puskesmas Putri Ayu, Jambi, Indonesia

**Keywords:** Psychological factors, COVID-19, Vaccine acceptance, Indonesia

## Abstract

**Introduction:**

Increasing the rate of vaccination is crucial in combating the COVID-19 pandemic. However, a survey of 112,888 Indonesians found that only 64.8% Indonesians were willing to be vaccinated, with 7.6% refusing all vaccines and 27.6% are unsure. Several factors were related to this vaccine hesitancy and refusal, such as cognitive reflection, trust in authoritative figures, and personality traits. This study aims to identify psychological determinants and other factors associated with vaccine hesitancy and vaccine refusal. This was a cross-sectional study with data collection done in March 2021 using a questionnaire. We collected demographic data, respondents' stance on vaccination, as well as their psychology measurement. IBM SPSS 26.0 (Statistical Package for the Social Sciences, IBM Corp., Armonk, NY, USA) was used for statistical analysis.

**Results:**

The data of 190 respondents were collected for this study. There are 165 respondents (86.8%) who belong to “vaccine acceptance”, while 25 are “vaccine hesitance” or “vaccine resistance.” Multivariate analysis shows that frequency of COVID-19 tests (*p* = 0.03), smoking status (*p* = 0.035), agreeableness trait (*p* = 0.001), trust in government (*p* = 0.04) and trust in scientist (*p* = 0.049) are significantly associated with the two population.

**Conclusion:**

Several demographic and psychological factors affect the COVID-19 vaccine acceptance. The government and other related parties should consider these factors when adjusting for future policies controlling the COVID-19 pandemic and increasing the vaccination rate.

## Background

Severe acute respiratory syndrome coronavirus 2 (SARS-CoV-2), which causes COVID-19, shows no signs of abating worldwide despite the pandemic inching closer to last 2 years [1]. While some government interventions are more successful than the others in curbing the virus spread [2], not all countries show equal success in controlling this pandemic [3].

An index of six criteria measuring confirmed cases, confirmed deaths, confirmed cases per million people, confirmed deaths per million people, confirmed cases as a proportion of tests, and tests per thousand people are created to compare how countries are doing in terms of their ranking globally. Out of 102 countries, Indonesia ranks 89th as of the 13th of March, 2021, dropping four places from 85th on the 9th of January, 2021 [4]. Despite numerous attempts and policies to prevent the spread of COVID-19, the number of cases keeps fluctuating wildly [3, 5].

One of the more prominent ways to combat the pandemic is ensuring Indonesians are fully vaccinated as soon as possible. This is seen from the rapidly changing guidelines and policies regarding who can be vaccinated and ensuring that the COVID-19 vaccine is readily available and distributed equally throughout all the provinces [6–8]. However, this program is not without any obstacles. Lack of trained medical staff, problems in cold-chain storage and distribution, financial issues, and vaccine refusal or vaccine hesitancy are some of the issues that slow down the COVID-19 vaccination program [9–11].

The government targets roughly 67% of Indonesians (181.5 million out of 270.9 million Indonesians) who should be vaccinated by the 31st of December, 2021 [12]. As of the 22nd of August 2021, 20.9% of Indonesians have received at least one jab, and 11.49% of Indonesians are fully vaccinated. Assuming there is no change in vaccination rate, Indonesia will hit a 70% fully vaccinated rate by the 21st of March, 2022 [13]. Encouraging Indonesians to be vaccinated is an issue. World Health Organization (WHO), Ministry of Health of the Republic of Indonesia, and United Nations' Children Fund (UNICEF) released a finding in November 2020. They found that out of 112,888 Indonesians surveyed, 64.8% were willing to be vaccinated, 7.6% refused all vaccines, and 27.6% were unsure [10].

Although numerous measures have been implemented to increase the rate of vaccination, such as expanding the target population and easier access to vaccination [8], vaccine hesitancy and vaccine refusal need to be addressed differently. Numerous psychological factors have been implicated in vaccine hesitancy and refusal, such as cognitive reflection [14], trust in the government, scientists, and healthcare professionals [15, 16], and personality traits [14]. The government and public health officials will know which demographics to target to improve the vaccine acceptance rate based on the psychological traits. Therefore, we aim to identify psychological determinants and other factors associated with vaccine hesitancy and vaccine refusal.

## Methods

We collected primary data from respondents directly through a two-part structured questionnaire. This was a cross-sectional study with purposive sampling. The first part consisted of demographic data collection, such as age, sex, race, marital status, comorbidities, highest education attained, monthly expenses, previous exposures or any close contact with COVID-19 patients, and whether respondents have any health insurance. The latter part of the questionnaire includes respondents' stance on vaccination before coming for a jab and their psychology measurement described below.

We included adults (> 18 years) who were vaccinated with CoronaVac (Sinovac Life Sciences, Beijing, China) in Puskesmas Putri Ayu, one of the biggest Puskesmas in Jambi city, Indonesia. Puskesmas are government-mandated community health clinics spread throughout Indonesia to promote primary prevention and healthier lives. Data collection was done from the 15th of March to the 25th of March 2021. Our exclusion criteria were broadly categorized into two, which were refusal to participate and contraindicated to COVID-19 administration. Due to the dynamic nature of clinical research and findings of COVID-19 vaccination, guidelines about who can be vaccinated were updated frequently, either by the government or Indonesian medical institutions. Therefore, we adhered to the Indonesian Society of Internal Medicine's recommendation (the 18th of March, 2021), which was the first to issue a recommendation about who could be vaccinated [15]. Patients with primary immunodeficiency, acute and active infections (including SARS-CoV-2 infections or 3 month post-infection), presented with a severe allergic reaction or anaphylaxis after the first dose of COVID-19 jab, blood pressure of ≥ 180/110 mmHg, unstable or uncontrolled chronic conditions, such as diabetes mellitus or heart failure, and those with Fatigue, Resistance, Ambulation, Illness, and Loss of weight (FRAIL) score of > 2 were contraindicated to COVID-19 vaccination. Although this recommendation specified that only 18–59 years should be vaccinated, on the 5th of February 2021, Indonesia's Food and Drug Administration issued an emergency use authorization that elderly (≥ 60 years) were eligible for vaccinations upon passing medical screenings [7]. Therefore, the elderly were also included in our study.

Respondents were classified according to their stance on COVID-19 vaccination. There was a question that went as follows: “Before coming to Puskesmas Putri Ayu, are you sure that you are ready to be vaccinated?” If respondents answered yes, they were classified as “vaccine acceptance”, no meant “vaccine-resistant”, and maybe meant that they were “accine-hesitant”.

Personality traits were assessed using The Big-Five Inventory (BFI-10). This inventory measured openness to new experiences, conscientiousness, extraversion, agreeableness, and neuroticism. Two items on a five-point Likert scale, ranging from “strongly disagree” (1) to “strongly agree”, are used to assess each attribute [16]. We used the translated and validated BFI-10 in the Indonesian language [17]. Internal reliability coefficients were not assessed because the scale only used two items to evaluate each personality trait. A study found that coefficient alpha was inaccurate for proving internal consistency in this situation [18].

We also assessed analytical or reflective reasoning with the help of The Cognitive Reflection Task (CRT), a three-item analytical reasoning test in which participants were asked to solve logical issues that imply intuitively attractive but erroneous answers [19].

Finally, respondents were asked to rate their trust in the government (which consists of the government itself, the state, and the parliament), scientists, physicians, and other health professionals. On a five-point Likert scale, responses ranged from “do not trust at all” (1) to “totally trust” (5) [20].

IBM SPSS 26.0 (Statistical Package for the Social Sciences, IBM Corp., Armonk, NY, USA, 2019) was used for statistical analysis. Normality testing was carried out using the Kolmogorov–Smirnov test, and if the *p* value is more than 0.05, the data had a normal distribution. Presentation of data using mean and standard deviation implied that data were distributed normally, while median and range meant not normally distributed.

Although previous studies have validated the internal reliability of the questionnaires, Cronbach's α application was specific to a particular sample of respondents [21]. Therefore, its internal reliability needed to be assessed in our population as well. Taber [22] classified Cronbach’s *α* value into several categories, such as: excellent (0.93–0.94), strong (0.91–0.93), reliable (0.84–0.90), robust (0.81), fairly high (0.76–0.95), high (0.73–0.95), good (0.71–0.91), relatively high (0.70–0.77), slightly low (0.68), reasonable (0.67–0.87), adequate (0.64–0.85), moderate (0.61–0.65), satisfactory (0.58–0.97), acceptable (0.45–0.98), sufficient (0.45–0.96), not satisfactory (0.4–0.55) and low (0.11).

There were five categories for income. Poor is defined as whose household expenses per month are less than Rp 1,416,000 (~ $99); vulnerable is defined as whose household expenses per month are between Rp 1,416,000 to Rp 2,128,000 (~ $99–$148); aspiring middle class is defined as whose household expenses per month are between Rp 2,128,001 to Rp 4,800,000 (~ $148 to $334); middle class is defined as whose household expenses per month are between Rp 4,800,001 to Rp 24,000,000 (~ $334 to $1671); and upper class is defined as whose household expenses per months are above Rp 24,000,000 (~ $1671) [10].

Bivariate analysis was done using chi-square, independent t-test when data distribution was normal, and Mann–Whitney when data distribution was not normal. When *p* values are below 0.25, those indicators are included in multivariate logistic regression analysis. The performance of our final prediction results would be checked for discrimination using receiver operating curve (ROC) and calibration (goodness of fit) using the Hosmer–Lemeshow test [23]. Area under the curve (AUC) will be interpreted from ROC. When the ROC curve corresponds to random chance, AUC would be equal to 0.5, and when the ROC curve corresponded to perfect accuracy, AUC would be 1.0 [24]. A good calibration would be measured by a *p* value of > 0.05 [25].

## Results

There are 190 participants in this study, with a predominance of female correspondents (54.2%) (Table 1). The mean age of the respondents is 44.84 years (SD ± 16.14), and most are married (70.5%). Most respondents fall into the category of the aspiring middle class (46.3%), with the majority having a bachelor's degree or higher (55.8%). Most of our respondents have never undergone any COVID-19 tests (56.3%), and 86.8% have no comorbidities. Most respondents register themselves for the vaccination program (57.4%), with 71.1% possessing national state insurance. Notably, there are four people (2.1%) who have a history of mental disorders. Among all the demographic criteria, only COVID-19 testing correlates significantly with vaccine acceptance or hesitance/resistance (*p* value of 0.048).

**Table 1:** Demographic characteristics of the respondents (*N* = 190)

Variable	*N*	%	*p* value
Sex			
Male	87	45.8	0.812
Female	103	54.2	
Age—mean (SD)	44.84	16.14	0.687
BMI—mean (SD)	24.17	4.04	0.516
Marriage status			
Single/divorced	56	29.5	1
Married	134	70.5	
Occupation			
Entrepreneur	60	31.6	0.228
Government worker	20	10.5	
Healthcare worker	12	6.3	
Housewife	19	10	
Religious leader	9	4.7	
Student/Jobless/Retired	44	23.2	
Teaching staff	26	13.7	
Monthly expenses			
< Rp. 1,416.000	25	13.2	0.273
Rp. 1,416,001–2,128,000	40	21.1	
Rp. 2,128,001–4,800,00	88	46.3	
Rp. 4,800,001–24,000,000	35	18.3	
> Rp. 24,000,000	2	1.1	
Highest education attained			
D3 or equivalent	12	6.3	0.265
Bacherlor’s/Master’s/Doctoral degree	106	55.8	
Primary school or equivalent	12	6.3	
Secondary school or equivalent	10	5.3	
High school or equivalent	44	23.1	
No formal education	3	1.6	
Did not finish primary school	3	1.6	
COVID-19 impact on occupation and income			
Income rises	2	1.1	0.344
Income drops by 50%	45	23.7	
No changes	47	24.7	
Currently not working	96	50.5	
Are there any close relatives that come in close contact with COVID-19 patients?			
No	155	81.6	0.213
Not sure	15	7.9	
Yes	20	10.5	
Who lives with you?			
Full family members	35	18.4	0.158
Some of the family members	88	46.3	
With my wife/husband only	46	24.2	
Alone	21	11.1	
Are there any kids in your house?			
No	90	47.4	0.777
Yes	100	52.6	
How many kids do you have? (*n* = 100)			
1	36	36	0.274
2	41	41	
> 3	23	23	
Have you done any COVID-19 tests before?			
No	107	56.3	0.048
Yes	83	43.7	
How many times have you done COVID-19 tests?			
1	42	22.1	0.08
2–5	34	17.9	
5–10	4	2.1	
> 10	3	1.6	
Never	107	56.3	
Comorbidities			
Yes	25	13.2	0.755
No	165	86.8	
Who registered you for the vaccination?			
Myself	109	57.4	0.365
Family members	40	21	
Close friend/Neighbor	41	21.6	
Do you have any history of mental disorders?			
No	186	97.9	0.434
Yes	4	2.1	
Do you smoke?			
No	171	90	0.027
Yes	15	7.8	
Have stopped < 5 years	2	1.1	
Have stopped ≥ 5 years	2	1.1	
Health insurance			
National state insurance	134	70.5	0.167
Private	5	2.6	
Both	36	18.9	
No health insurance	15	8	

There are 165 respondents (86.8%) who belong to “vaccine acceptance”, while 25 respondents (13.2%) are “vaccine hesitance” or “vaccine resistance”. Amongst the personality traits, openness scores the highest with a mean score of 32.9 (SD ± 4.55) in the vaccine acceptance group and 30.64 (SD ± 6.32) in the vaccine hesitance and vaccine resistance group (Table 2). Respondents score poorly on CRT with a mean score of 0.31 (SD ± 0.69) and 0.20 (SD ± 0.58) in the vaccine acceptance and hesitance and resistance groups, respectively. Although the trust in government has the highest score, this number cannot be directly interpreted. This is a combination of trust in three combined institutions (the state, the government, and the parliament). Agreeableness (*p* < 0.001), neuroticism (*p* = 0.012), trust in the government (*p* = 0.005), trust in scientist (*p* = 0.010), and trust in health care professionals (*p* = 0.029) possess significant correlations towards two populations studied.

**Table 2:** Physiological indicators amongst vaccine acceptance, vaccine hesitancy, and vaccine resistance

	Vaccine Acceptance (*n* = 165)			Vaccine hesitance and resistance (*n* = 25)			*p* value
	Mean	SD	SE	Mean	SD	SE	
Personality							
Extraversion	27.05	3.46	0.27	25.76	3.98	0.80	0.091
Agreeableness	28.57	4.04	0.31	24.48	3.70	0.74	< 0.0001
Conscientiousness	26.70	3.55	0.28	25.32	2.91	0.58	0.065
Neuroticism	25.32	3.92	0.31	23.12	4.61	0.92	0.012
Openness	32.90	4.55	0.35	30.64	6.32	1.26	0.097
Cognitive reflection task							
Test 1–3	0.31	0.69	0.05	0.20	0.58	0.12	0.451
Trust							
Government^a^	10.32	1.85	0.14	8.20	3.37	0.67	0.005
Scientist	3.69	0.72	0.06	3.04	1.14	0.23	0.010
Health care professionals	3.81	0.75	0.06	3.20	1.29	0.26	0.029

^a^Includes the state, the government, and the parliament combined

**Fig. 1:**
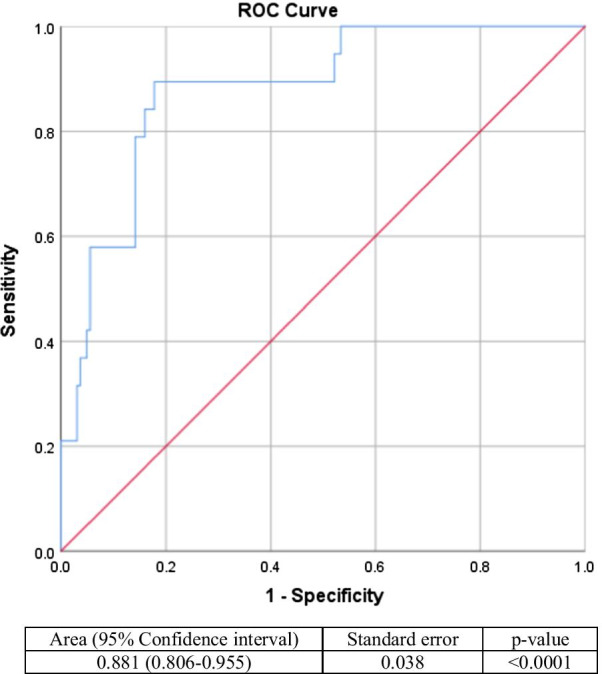
Receiver operating curve to assess discrimination of the model

Table 3 shows the multivariate analysis, and it shows that the more frequently a respondent tests for COVID-19, he or she is more likely to be in the vaccine acceptance group with an odds ratio of 0.13 (95% CI 0.03–0.5; *p* value 0.03). Similarly, smokers are more likely to be in the vaccine acceptance group with an odds ratio of 0.24 (95% CI 0.064–0.9; *p* value 0.035). Respondents who score higher on the agreeableness category are also more inclined to be in the vaccine acceptance group with an odds ratio of 0.74 (95% CI 0.62–0.89; *p* value 0.001). Finally, the more trust a respondent puts in the government and scientists, the more likely he or she belongs to the vaccine acceptance group with an odds ratio of 0.7 (95% CI 0.5–0.98; *p* value 0.04) and 0.4 (95% CI 0.14–0.6; *p* value 0.049), respectively. Hosmer–Lemeshow test shows that this model is a good fit with a *p* value of 0.619 (results not shown). The AUC for this model is 0.991 (95% CI 0.806–0.955; *p* value < 0.0001) (Fig. 1), which shows that this model has good discrimination.

**Table 3:** Multivariate analysis of demographic factors and physiological indicators amongst vaccine acceptance, vaccine hesitancy, and vaccine resistance

Variables	Multivariate logistic analysis	
	Odds ratio (95% CI)	*p* value
Demographic data		
Occupation	1.33 (0.98–1.8)	0.061
Frequency of COVID-19 tests	0.13 (0.03–0.5)	0.03
Smoking status	0.24 (0.064–0.9)	0.035
Health insurance	0.45 (0.19–1.05)	0.067
Personality		
Extraversion	–	–
Agreeableness	0.74 (0.62–0.89)	0.001
Conscientiousness	–	–
Neuroticism	–	–
Openness	–	–
Cognitive reflection task		
Test 1–3	–	–
Trust		
Government^a^	0.7 (0.5–0.98)	0.04
Scientist	0.4 (0.14–0.6)	0.049
Health care professionals	–	–

^a^Includes the state, the government, and the parliament combined

## Discussion

The majority of our study respondents belong to the vaccine acceptance group. This result is consistent with a COVID-19 vaccine acceptance survey in Indonesia, where most Indonesians were in the vaccine acceptance group. In Jambi, where the population of our studies was taken, the national survey reported a 65% vaccine acceptance rate [10]. Although the survey was done 6 months apart, their acceptance rate is still similar.

Our studies found that females were more likely to be in the vaccine acceptance group. These findings are in line with other studies that stated females are more likely to accept vaccines [26]. Compared to a survey in Indonesia, the acceptance rate between male and female respondents is almost the same at 65%. However, less than 5% of females would refuse a vaccine, while 10% of males would refuse a vaccine [10]. The age findings in our study were consistent with the Indonesian government policy when data gathering was conducted, thus explaining the mean age were on the younger side compared to a study from the United States, where most of the respondent is in the 50–64-year-old range [27]. Respondents who are married are more likely to be vaccinated due to the urge to protect their partners. Married people also tend to have more awareness about vaccines than unmarried counterparts due to frequent information sharing between partners [28]. Those who fall in the middle class would prefer to continue working to gain money. At the same time, those with bachelor's degrees or higher are also more likely to accept vaccines due to a better understanding of vaccine efficacy and safety. This finding is similar to another study [29], where those with higher education status are more likely to be vaccine acceptance.

Even though those who have done COVID-19 tests are significantly associated in the bivariate analysis, the multivariate analysis showed only the frequency of COVID-19 tests and smoking status are significantly associated with vaccine acceptance in the demographic criteria. Those with a higher perceived risk of being infected with COVID-19 tend to do more preventive measures, such as doing more COVID-19 tests, and are more likely to accept the COVID-19 vaccine because of their better awareness of the scope of this pandemic and fear of getting infected [30, 31]. Smokers tend to suffer from severe COVID-19, and thus, they have a better understanding that they are more prone to a worse prognosis. Furthermore, smoking is found to reduce vaccine efficacy. Taken altogether, these reasons might explain why smokers are more likely to be in the vaccine acceptance group [32, 33].

Other studies from the United States, Ireland, and the United Kingdom share similar agreeableness traits. People who possess this trait are significantly associated with COVID-19 vaccine acceptance and vaccination in general. This may be due to their optimistic views and belief that the vaccine is a beneficial invention for humanity [20, 34].

We found that trust in government and scientists is significantly associated with vaccine acceptance and concurrence with various studies [20, 35, 36]. Governments and experts, including scientists, play a crucial role in determining the vaccine acceptance of its nations in this pandemic situation through their policy makings and scientific breakthroughs [37]. Unfortunately, a series of denial, reluctance, and refusal has delayed Indonesia's response towards the COVID-19 crisis, which ultimately increases distrusts amongst Indonesians towards their governments [37]. As for scientists, the main problem is a lack of communication towards the general population regarding current COVID-19 developments and vaccinations, as well as concerns regarding scientists' personal bias and corporate agendas, which may cause Indonesians to lose trust in our country's scientists [38].

One study shows that even subjects with mental disorders show a higher aptitude and willingness to pay for the COVID-19 vaccine. The same study also finds that having private health insurance and living with children or dependents are associated with a higher willingness to be vaccinated. At the same time, these findings are not significant in our study [39]. Another study looking at healthcare workers in Asia–Pacific finds that 95% of the respondents are willing to be vaccinated, in contrast to 86.8% in our study [40]. However, some considerations should be taken into account when accounting for the higher rate of vaccination in healthcare workers, such as mandate bias by the institutions or state [41, 42], fear of contracting the virus as a frontline worker [43], and attitudes towards vaccination [44]. Gauging vaccine acceptance in healthcare workers also needs further research as results are still conflicting as to whether healthcare workers embrace or oppose COVID-19 vaccination [39, 40, 44–47].

There are a few limitations to our study. First of all, during data collection, there are a few vaccination policies changed by the government. This might introduce a population bias in our study, where only the selected age population is included during this policy. Second, not every psychological domain could be studied due to the limited time of filling the questionnaire. Third, the potential of a collider bias exists and hence undermines the results of our study [48]. Fourth, some populations are not included in our study, such as pregnant women, as the guideline did not yet recommend vaccination on this population [49]. Last, our study sample could not represent the whole Indonesian population, because our data collection was conducted only in a single vaccination centre in Jambi.

Despite the limitations, our paper also has its strengths. First of all, our study is one of the first few studies that analyze psychological factors that could affect vaccine acceptance in Indonesia. This provides principal results in adjusting further government policies to ensure vaccine administration remains high. The government and scientists should work together to implement new policies that focus on regaining people's trust so that people who are “vaccine-hesitant” or “vaccine refusal” will consider taking a jab. By identifying and applying ethical and procedural principles of vaccination, the rate of COVID-19 vaccination will be significantly boosted [50]. Our study achieves a good Hosmer–Lemeshow test and AUC results which means that this predictive model possesses good calibration and discrimination.

## Conclusions

In conclusion, the psychological factor is an essential factor that affects COVID-19 vaccination. Our study found that the frequency of COVID-19 tests, smoking status, agreeableness personality trait, and trust in government and scientists are significantly associated with vaccine acceptance with good predictive factors and discriminant. Thus, this model could be used as a basis for health care providers, government, scientists, and other parties to convince those who are hesitant and resistant towards the COVID-19 vaccine into being vaccine acceptance.

## Data Availability

Available upon request.

## Abbreviations

SARS-CoV-2: Severe acute respiratory syndrome coronavirus 2
COVID-19: CoronaVirus Disease-2019
WHO: World Health Organization
UNICEF: United Nation's Children Fund
BFI-10: Big-Five Inventory
CRT: Cognitive Reflection Task
ROC: Receiver operating curve
AUC: Area under the curve
SPSS: Statistical Package for the Social Sciences
